# Low depression literacy exacerbates the development and progression of depressive mood in Chinese adult social media users during COVID-19: A 3-month observational online questionnaire-based study with multiple cross-sectional analyses

**DOI:** 10.3389/fpubh.2023.1096903

**Published:** 2023-02-16

**Authors:** Dan Shan, Shaoyang Li, Ruichen Xu, Jingtao Huang, Yi Wang, Yuandian Zheng, Shanshan Huang, Yuming Song, Junchu Han, Sayaka Suto, Zhihao Dai

**Affiliations:** 1 COVID-19 Response Social Organization Collaboration Network, Qujing, China; ^2^Faculty of Science, The Hong Kong Polytechnic University, Kowloon, Hong Kong SAR, China; ^3^Department of Integrative Biology, University of Wisconsin-Madison, Madison, WI, United States; ^4^School of Basic Medical Science, Capital Medical University, Beijing, China; ^5^China-Japan Friendship Clinical School, Beijing University of Chinese Medicine, Beijing, China; ^6^Department of Medical Imaging, Qujing Second People's Hospital of Yunnan Province, Qujing, China; ^7^School of Medical Imaging, Hebei Medical University, Shijiazhuang, China; ^8^Department of Human Development, Columbia University, New York, NY, United States; ^9^School of Medicine, Royal College of Surgeons, Ireland, University of Medicine and Health Sciences, Dublin, Ireland

**Keywords:** Chinese adults, COVID-19, depression, depression literacy, mental health

## Abstract

**Background:**

The main purpose of this study was to explore the relationship between depression literacy (D-Lit) and the development and progression of depressive mood.

**Methods:**

This longitudinal study with multiple cross-sectional analyses used data from a nationwide online questionnaire administered *via* the Wen Juan Xing survey platform. Eligible participants were 18 years or older and had subjectively experienced mild depressive moods at the time of their initial enrollment in the study. The follow-up time was 3 months. Spearman's rank correlation test was used to analyze the predictive role of D-Lit on the later development of depressive mood.

**Results:**

We included 488 individuals with mild depressive moods. No statistically significant correlation between D-Lit and Zung Self-rating Depression Scale (SDS) at baseline was observed (adjusted rho = 0.001, *p* = 0.974). However, after 1 month (adjusted rho = −0.449, *p* < 0.001) and after 3 months (adjusted rho = −0.759, *p* < 0.001), D-Lit was significantly and negatively correlated with SDS.

**Limitations:**

The targeted subjects were limited to the Chinese adult social media users; meanwhile, China's current management policies for COVID-19 differ from most of the other countries, limiting the generalizability of this study.

**Conclusion:**

Despite the limitations, our study provided novel evidence supporting that low depression literacy may be associated with exacerbated development and progression of depressive mood, which, if not appropriately and promptly controlled, may ultimately lead to depression. In the future, we encourage further research to explore the practical and efficient ways to enhance public depression literacy.

## 1. Introduction

### 1.1. Prevalence of depression in China during COVID-19 pandemic

Depression has always been a major public health concern that places a significant economic and emotional burden on society (1), particularly exacerbated during the pandemic era (2, 3). Pre-pandemic National Household Surveys noted that 38% of Chinese adults experienced depressive symptoms and approximately 4% suffered from depression (4, 5). In the early stage of the COVID-19 pandemic, the national prevalence of depression increased to 6.3% (6), while this trend further rose to more than 15% during the peak of the COVID-19 pandemic in China (7).

### 1.2. Depression and its relevant risk factors

Although the pathogenesis of depression is still under exploration, the previous study has found many risk factors that are associated with the occurrence and development of depression (8). The most prevalent factors identified during the pandemic era are chronic anxiety (9), low socio-psychological status (10), low health literacy (11), major life-changing events (12), job insecurity (13), and history of other psychiatric disorders (14).

### 1.3. Depression and depression literacy

Mental health literacy (MHL), derived from health literacy, is defined as a person's ability to recognize, manage, and prevent mental illness (e.g., knowing how and where to seek help when needed) (15). Depression literacy (D-Lit), a type of MHL that focuses explicitly on depression, is primarily defined as a person's knowledge reservation toward depression (16–18). People with high D-Lit are more likely to proactively seek professional help with more optimistic mindsets about their mental problems induced by depressive disorder (19). Meanwhile, people with inadequate MHL are significantly more likely to have diagnoses of depression in comparison with those with sufficient MHL (20–22), not excepting during COVID-19 (23).

### 1.4. The present study

In the current pandemic era, the number of people suffering from depression or depressive symptoms has increased dramatically. Hence, it is critical to find practical approaches to reflect an individual's depressive state and prevent it from deteriorating into a major depressive disorder at the earliest stage. Although cumulative studies have examined the association between low MHL or low D-Lit and an increment in depression likelihood (11, 20–22, 24), scant previous research has investigated the role of D-Lit in facilitating the development and progression of an individual's initial depressive state that ultimately deteriorates into depressive disorder. Goldney et al. pointed out the importance of MHL for the optimal management of depression in its early stages (11, 25). Similarly, Yang et al. suggested that enhancing self-D-Lit could moderate early precursors of later depression disorder by facilitating earlier access to appropriate mental health care (19).

Thus, this study was mainly intended to do the following:

1. Roughly ascertain the status of depression literacy in Chinese adult social media users.
2. Identify the predictive role of D-Lit (if any) in subsequently potential development and progression of depressive mood, especially in the context of COVID-19, among these Chinese adult social media users.
3. Explore the relationship between D-Lit and Chinese adults' proactive adoption of protective behaviors against depression mood, as the D-Lit questionnaire does not directly ask questions about this aspect. The protective behaviors here mainly refer to help-seeking behaviors.

The findings of this study may have implications for early intervention in Chinese adult social media users prone to depressive symptoms *via* an enhancement of depression literacy, thereby reducing the probability of developing depression later in life.

### 1.5. Research hypotheses

Based on the previous cumulative evidence on the cross-sectional relationship between MHL or D-Lit and depression, we came up with the following hypotheses:

***Hypothesis 1:*** Early low depression literacy predicts the later development and progression of depression in Chinese adults while controlling for other factors.

***Hypothesis 2:*** Depression literacy among Chinese adults positively predicts their proactive protective behaviors (e.g., help-seeking behavior) performed to mitigate the negative impact of depressive symptoms on themselves.

## 2. Materials and methods

### 2.1. Overview

We conducted a longitudinal quantitative study with multiple cross-sectional analyses based on online questionnaire surveys from July 2022 to October 2022. Primary inclusion criteria for intended participants emphasized that they should be Chinese adults (≥18 years old) suffering from mild low mood at the time of recruitment. Data were collected nationwide randomly and anonymously *via* a professional data collection platform—Wen Juan Xing (Changsha Ranxing Information Technology Co., Ltd., Hunan, China), which is very straightforward and friendly for users (i.e., potential participants). Its validity for conducting online survey studies has been verified in previous studies (26–28). The use of human data from the surveys was carried out in an ethical manner, in line with the principles of the Declaration of Helsinki (as revised in 2013) (29). On the first page of the survey, all respondents received an accurate description of the survey purpose and were asked to confirm their online informed consent before continuing the survey. All participants were asked to complete three questionnaire surveys longitudinally in July, August, and October 2022, where possible, according to their personal circumstances and lives. They were asked to complete a comprehensive questionnaire at the beginning of this observational experiment (i.e., in July 2022). Subsequently, our two follow-up surveys in August and October 2022 included only the self-rating depression scale (SDS) and a specific question about whether they experienced any personal event that acutely and severely worsened their mood (refer to the subsection of “questionnaire contents” below). At the end of the experiment, ideally, all participants would have completed the evaluations of the self-rating depression scale three times at the aforementioned three time points and the D-Lit questionnaire once in July 2022. Additionally, to ensure the quality of participants' responses, an attention check question was embedded in the questionnaire at each time of their response (e.g., “At what age is a person considered an adult in China?”, “Please select the answer Orange”).

### 2.2. Questionnaire contents

The questionnaire mainly contained the following information collected:

1. Demographic information of participants.
2. “Whether you had any past physician-confirmed history of psychiatric disorders?” Psychiatric disorders here can refer to anxiety disorder, depressive disorder, bipolar disorder, post-traumatic stress disorder (PTSD), schizophrenia, eating disorders, etc.
3. “Whether you were experiencing a mild depressive mood in recent several days?”
4. “To what extent are you willing to take protective behaviors (e.g., actively seek help from a clinical psychologist or psychiatrist) to combat depressive mood?”
5. Depression Literacy Questionnaire (D-Lit), 22-item version.
6. Zung Self-rating Depression Scale (SDS), 20-item version.
7. “Did you experience any personal events during the survey period that led to a significantly acute deterioration in your mood?”

Finally, 600 Chinese adults nationwide were recruited and surveyed through the Wen Juan Xing platform based on the G^*^Power 3.1 software for sample size estimation (30). Of them, 34 were excluded for the failure in the attention check questions (e.g., responded wrongly to the instruction “please choose the answer Orange”); 28 were excluded for the absence of follow-up surveys; 27 were excluded for completing the survey in less than 200 s; 11 were excluded for significantly acute deterioration in their mood due to unexpected personal events during the follow-up survey period; and 12 were excluded with additional analyses for other reasons such as answering the questionnaire questions inconsistently or contradictorily. Eventually, a valid sample of 488 participants was analyzed collectively (251 women and 237 men; mean age = 32.62 years, SD = 6.71 years; age range: 18–49 years). The effective response rate was 81.3%.

### 2.3. Depression literacy assessment tool

The participants' depression literacy (D-Lit) was evaluated using the 22-item version of the D-Lit Questionnaire. It measures the level of mental health literacy of people specific to depression. The questions in the questionnaire are rated by the respondents on a three-point scale of “true” “false,” and “do not know.” One point is awarded for each correct response; therefore, higher scores indicate a higher level of personal health knowledge about depression (total score ranges from 0 to 22) (31). In Griffiths' study, the validity and reliability of this instrument were assessed based on a sample of elite athletes, with Cronbach's alpha and test–retest reliability of 0.70 (*n* = 40) and 0.71 (*n* = 12), respectively (31). Taking into account translation and acculturation factors, Wang et al. confirmed the reliability of the Chinese version of the questionnaire with Cronbach's alpha coefficient of 0.885 (32). Cronbach's alpha coefficient for the scale in the current study was 0.84, indicating excellent internal consistency.

### 2.4. Depressive tendency assessment tool

The Zung Self-rating Depression Scale (SDS) is a self-reported 20-item Likert-style questionnaire, each rated on a four-point scale. It is widely used to measure the depressive tendencies of respondents. Little to no depression, mild depression, moderate depression, and severe depression range from 0 to 44, 45 to 59, 60 to 69, and 70 to 80, respectively (33). The reliability and validity of the Chinese version of SDS were confirmed by Lee et al., with Cronbach's alpha coefficient of 0.91 and Guttman's split-half reliability of 0.89 (34). Cronbach's alpha coefficient for the scale in the current study was 0.88, indicating excellent internal consistency.

### 2.5. Statistical analysis

All statistical analyses were performed using the software program SPSS (version 25.0), except for the data cleaning process, which included detecting and removing invalid or missing data on Excel. Reliability tests were conducted for the D-Lit Scale and Zung SDS, using Cronbach's alpha coefficients to measure internal consistency (α > 0.70 regarded as acceptable). For all parameters considered for comparison in the study, the different statistics calculated in Table 1 (e.g., skewness and Shapiro–Wilk test) suggested the skewed distribution of data, thus yielding the non-parametric analyses in Table 2. Data were expressed as the median and interquartile range (IQR). The Mann–Whitney U or Kruskal–Wallis non-parametric tests were used to assess the differences in mean ranks of D-Lit scores and SDS scores at various stages concerning gender, age group, marital status, highest educational level, financial status, and prior physician-confirmed psychiatric history. Spearmen's correlation was applied to examine the predictive role of participants' D-Lit scores on the subsequent development and progression of depressive mood (i.e., Zung SDS scores) at baseline, after 1 month, and 3 months, respectively. Statistical significance was defined as a *p*-value of < 0.05.

**Table 1:** Normality tests.

**Statistics**	**D-Lit score**	**Zung SDS score**		
		**At baseline**	**1 month later**	**3 months later**
*n*				
Valid	488	488	488	488
Missing	0	0	0	0
Mean	8.950	47.800	44.024	39.720
Standard deviation	2.639	6.841	6.456	10.982
Skewness	0.600	−0.868	0.472	1.003
SE of skewness	0.111	0.111	0.111	0.111
Kurtosis	0.526	2.403	2.611	1.346
SE of kurtosis	0.221	0.221	0.221	0.221
Shapiro-wilk test's sig value	< 0.001	< 0.001	< 0.001	< 0.001

D-Lit, depression literacy; Zung SDS, Zung self-rating depression scale; Sig, significance.

**Table 2:** Non-parametric assessments of demographic factors (*n* = 488).

**Variables**		***n* (%)**	**Zung SDS score median (IQR)**			***p*-value**	**D-Lit median (IQR)**	***p-*value**
			**Baseline**	**1 month later**	**3 months later**			
Gender	Male	237 (48.6)	49 (46–52)	43.5 (40–48)	39 (30–45)	>0.05^a^	9 (7–11)	0.738^a^
	Female	251 (51.4)	49 (45–52)	44 (40.5–47)	39 (32–44)		9 (7–10)	
Age group	18–30	346 (70.9)	49 (45–52)	44 (40–47.5)	39 (30–45)	>0.05^b^	9 (7–10)	0.705^b^
	31–39	128 (26.2)	50 (46–52)	43.5 (40.626–46)	39 (32–43.75)		9 (7–10)	
	40–49	14 (2.9)	50 (47–53)	44.5 (39.75–50.5)	35 (27.75–47)		9 (7.25–12.25)	
Marital status	Single	161 (33)	49 (46–52)	44 (40.25–47.5)	39 (30–45)	>0.05^a^	9 (7–10)	0.805^a^
	Married	327 (67)	50 (45–52)	44 (40–47)	39 (31–44)		9 (7–10)	
Highest educational level	Illiteracy	0 (0)	N/A	N/A	N/A	>0.05^b^	N/A	0.442^b^
	Elementary	9 (1.8)	49 (46–52.5)	46 (38.75–51)	40 (27.5–57.5)		9 (7–11)	
	High school	50 (10.2)	50 (45–52)	42.5 (38.375–48)	37 (30–45)		8 (7–11.25)	
	College	316 (64.8)	49 (45–52)	44 (40–47)	39 (30–44)		9 (7–10)	
	Postgraduate	113 (23.2)	50 (46–52)	44.5 (41–49)	39 (34–49)		8 (7–10)	
Financial status	Weak	25 (5.1)	49 (40–52)	45.5 (36.25–48.5)	41 (35.5–51)	>0.05^b^	8 (6–9)	0.192^b^
	Medium	409 (83.8)	49 (46–52)	44 (40–47.5)	39 (30–44)		9 (7–10)	
	Good	54 (11.1)	49 (45–52)	44.25 (41.88–46.25)	38.5 (35–46.25)		9 (7–10)	
Psychiatric history	No	439 (90)	49 (46–52)	43.5 (40–47)	39 (30–44)	< 0.001^a^ ^c^	9 (7–11)	< 0.001^a^
	Yes	49 (10)	50 (45.5–52)	48 (44.75–55)	50 (40–67)		7 (4–8.5)	
Initiatives in taking protective behaviors	Never	59 (12.1)	49 (46–51)	47 (43.5–52)	47 (40–55)	< 0.001^b^	7 (6–8)	< 0.001^b^
	Sometimes	338 (69.3)	49 (45.75–52)	44.5 (41.5–47.5)	40 (36–45)		8 (7–9)	
	Always	91 (18.6)	50 (45–52)	39 (35–43)	28 (25–29)		12 (11–14)	

^a^Mann–Whitney *U* test.

^b^Kruskal–Wallis H test.

^c^denotes that the results based on this factor were not significant at baseline (*p* = 0.439), but strongly significant after 1 (*p* < 0.001) and 3 months (*p* < 0.001). D-Lit, depression literacy; Zung SDS, Zung self-rating depression scale.

## 3. Results

### 3.1. Survey participants' profile

The prevalence of depression (SDS score ≥ 45) in the current study was 85% at baseline, 43% after 1 month, and 25% after 3 months, respectively. Of the 488 survey participants, 237 (48.6%) were men, and 251 (51.4%) were women. The ages of these participants ranged from 18 to 49 years. The majority of participants (*n* = 346, 70.9%) belonged to the 18–30 age group, followed by those aged 31–39 years (*n* = 128, 26.2%) and 40–49 years (*n* = 14, 2.9%). Regarding marital status, most participants (*n* = 327, 67%) were married, followed by unmarried single (*n* = 161, 33%). In terms of the highest level of education, the majority of these participants completed college (*n* = 316, 64.8%), followed by postgraduate (*n* = 113, 23.2%), high school (*n* = 50, 10.2%), elementary (*n* = 9, 1.8%), and illiteracy (*n* = 0, 0%). As far as financial status is concerned, most of the participants belonged to the medium level (*n* = 409, 83.8%), followed by good (*n* = 54, 11.1%), and then weak (*n* = 25, 5.1%). In terms of previous physician-confirmed psychiatric history, 439 (90%) participants did not have such experience, while 49 (10%) did. Finally, for participants' proactivity in protective behaviors toward depressive mood, most participants did so sometimes (*n* = 338, 69.3%), followed by always (*n* = 91, 18.6%), and then never (*n* = 59, 12.1%) (refer to Table 2).

### 3.2. Differences in mean ranks in Zung SDS at different time points and D-Lit based on various variables

Among all the variables examined, no statistically significant differences in mean rankings were found for gender, age group, marital status, the highest level of education, and financial status (all *p* > 0.05). However, participants with a psychiatric history had lower mean rankings on the D-Lit than those without psychiatric history (131.64 vs. 257.10, *p* < 0.001), who had lower mean rankings on the Zung SDS scores after 1 (232.84) and 3 months (231.71), in comparison with those with psychiatric history (348.95 and 359.07, *p* < 0.001). Participants who always proactively engaged in protective behaviors to combat depressive mood had higher mean rankings on D-Lit (417.93, *p* < 0.001) and lower rankings on the Zung SDS after 1 and 3 months (139.66 and 84.31*, p* < 0.001), as compared to the participants who only occasionally (217.87, 258.21, and 268.07) and never (129.56, 327.67, and 356.53) engaged in these behaviors (refer to Table 2).

### 3.3. Spearman's correlation between D-Lit of and SDS at baseline, after 1 month, and after 3 months, as well as protective behaviors

**Figure 1:**
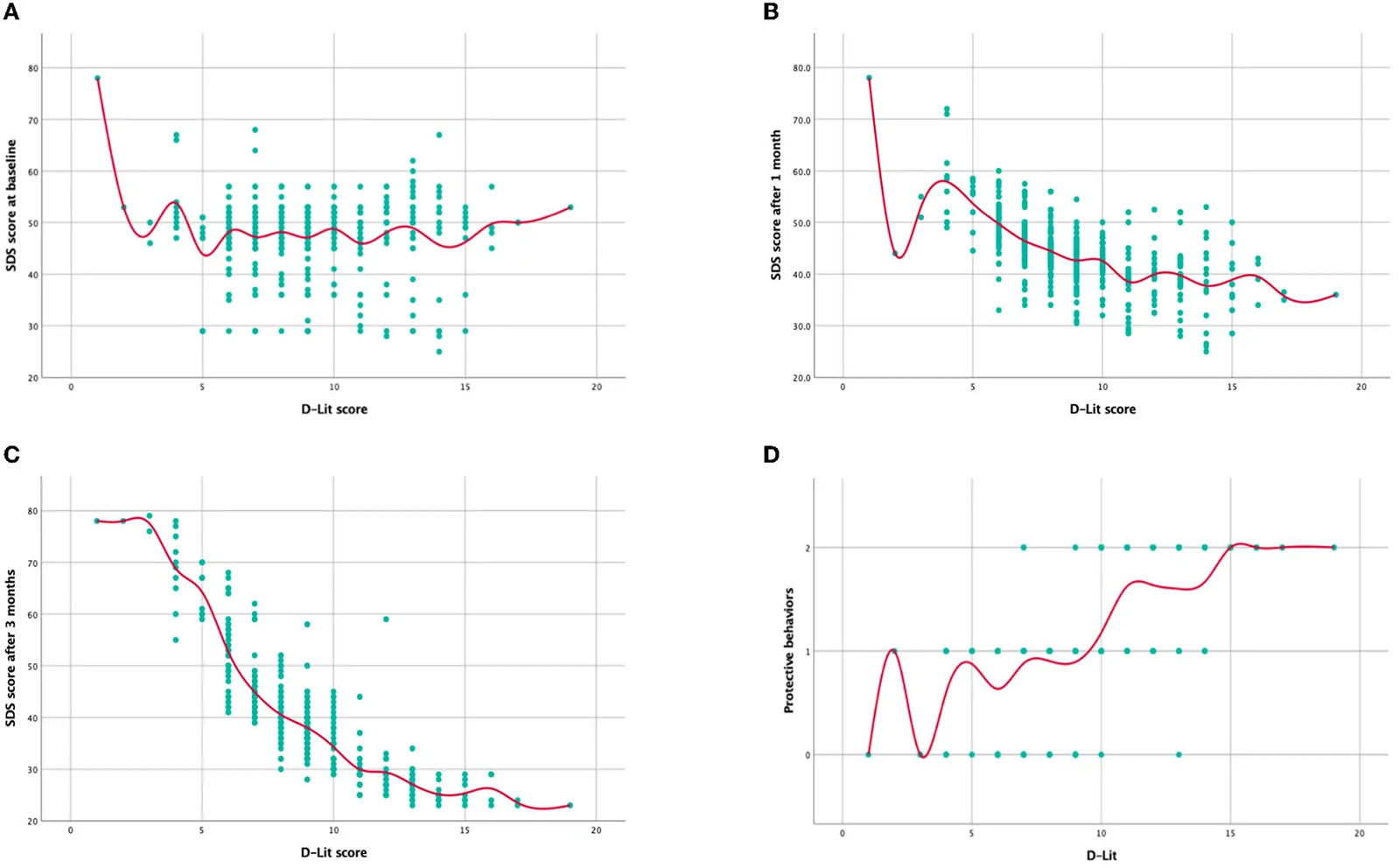
The correlation between D-Lit and SDS at different time points, and protective behaviors of participants. **(A)** Correlation between D-Lit score and Zung SDS score at baseline (Spearman's correlation test, statistical significance *p* = 0.344). **(B)** Correlation between D-Lit score and Zung SDS score after 1 month (Spearman's correlation test, statistical significance *p* < 0.001). **(C)** Correlation between D-Lit score and Zung SDS score after 3 months (Spearman's correlation test, statistical significance *p* < 0.001). **(D)** Correlation between D-Lit and protective behaviors of participants against depressive mood (Spearman's correlation test, statistical significance *p* < 0.001). In the y-axis of picture D, 0 denotes “Never” 1 denotes “Sometimes” and 2 denotes “Always.” D-Lit, depression literacy; SDS, self-rating depression scale.

Regarding Spearman's correlations (refer to Table 3), it showed that D-Lit scores had significant negative correlations with Zung SDS scores after 1 month (Figure 1B, unadjusted rho = −0.620, *p* < 0.001; adjusted rho = −0.449, *p* < 0.001) and after 3 months (Figure 1C, unadjusted rho = −0.888, *p* < 0.001; adjusted rho = −0.759, *p* < 0.001). D-Lit scores had a significant positive correlation with participants' proactivity in protective behaviors against depressive mood (Figure 1D, unadjusted rho = 0.607, *p* < 0.001; adjusted rho = 0.621, *p* < 0.001). No statistically significant correlation between D-Lit and SDS at baseline was observed (Figure 1A, unadjusted rho = 0.043, *p* = 0.344; adjusted rho = 0.001, *p* = 0.974).

**Table 3:** Statistical correlation between D-Lit of participants and Zung SDS at baseline, after 1 month, after 3 months; and proactive attitudes of participants in performing protective behaviors against depressive mood.

**Variable**	**Figure**	**Correlation**	**Spearman's coefficient**	**Significance**
**D-Lit**	1A	SDS at baseline	0.043 (Full)	0.344
			0.001 (Partial/Weighted)	0.974
	1B	SDS after 1 month	−0.620 (Full)	< 0.001
			−0.449 (Partial/Weighted)	< 0.001
	1C	SDS after 3 months	−0.888 (Full)	< 0.001
			−0.759 (Partial/Weighted)	< 0.001
	1D	Protective behaviors	0.607 (Full)	< 0.001
			0.621 (Partial/Weighted)	< 0.001

## 4. Discussion

The primary objective of this study was to explore the potential association between depression literacy and the development and progression of depressive mood. We also analyzed the association between depression literacy and participants' proactivity to engage in protective behaviors against depressive mood in Chinese adults during COVID-19. Participants' Zung SDS scores were collected at different time points (i.e., at baseline, after 1 month, and after 3 months) to reflect the progression of depressive mood. According to the results of the study, there were no significant differences in D-Lit and SDS scores among the participants of different genders, age groups, marital status, the highest level of education, and financial status, which were consistent with two previous studies (35, 36). However, this finding contradicts two other previous studies that concluded that people with higher education levels and female gender tended to have higher depression literacy (37, 38). These differences may be caused due to the nature of different samples.

We found that participants with a history of psychiatric disorders tended to have lower depression literacy and higher SDS scores compared to those without it. Participants with a relatively higher D-Lit were more likely to proactively take protective behaviors to combat depressive moods. Meanwhile, participants who were more proactive in protective behaviors to combat depressive mood were likelier to have lower SDS scores than those who never took protective behaviors. Most importantly, participants' D-Lit could predict their subsequent depressive progression negatively. We observed that participants with lower D-Lit had relatively significantly higher SDS scores over time, and this negative correlation was most pronounced after 3 months. The same associations remained when controlling for other variables (i.e., gender, age group, marital status, the highest level of education, financial status, psychiatric history, and protective behavior activism), indicating that lower D-Lit might be an independent risk factor for the development and progression of depressive symptoms. Overall, these results were in line with our hypotheses and previous studies (21, 22, 36, 39–42).

These consistent findings can be interpreted in terms of prevention, early diagnosis, and early treatment of depression. People with high depression literacy reflect a better understanding of depression, so that they can constantly monitor their emotional changes to tell whether they are predisposed to depressive symptoms. Even if they are suffering from depressive moods as a result of an unexpected psychologically distressing event, they can recognize depressive signs and symptoms at a very early stage. This means that they can apply various approaches to promptly manage their low mood. Second, resilient attitudes toward depression have been reported to be consistently effective in combatting depressive symptoms (43). People with high depression literacy usually possess a positive mindset toward depression, which helps them cope with stress, anxiety, and depression.

In contrast, people with low depression literacy have a pessimistic attitude toward depression. For instance, many people with low depression literacy diagnosed with mild or moderate depression do not believe that their depression can be reversible, so they give up trying any solution against it. This pessimistic attribution style can often elicit negative implications in their daily life and impact social relationships (44). As a result, their depressive state deteriorates to a severe stage over time. Third, it is essential to note that when self-management of depressive mood does not work, it is better to seek help from externally reliable sources. People with high depression literacy are more likely to timely seek appropriate help from professional sources (e.g., finding a clinical psychologist or psychiatrist) when depressive symptoms appear in the earliest stages to fight against depressive mood and therefore prevent its further deterioration. In contrast, people with low depression literacy tend to perceive depression as a social stigma and fear of seeking external help (45), ultimately accelerating the development and progression of depression.

Theoretically, individuals who report having a history of psychiatric disorders could possess higher D-Lit compared with those who do not, as these individuals learn more about depression over the course of their long-term treatment. This trend has been shown in previous studies (18, 46). However, this was not the case in our finding in this regard. The potential explanation for this aspect could be due to two reasons. First, individuals with a history of psychiatric disorders do not necessarily imply that they have a history of depression. Hence, these individuals may not be as well-informed about depression as might be expected. Another possible reason is that patients with a history of psychiatric disorders may have negatively obstinate mindsets toward depression. People with a history of depression who now have a high D-Lit and thus know what and how to deal with depressive symptoms in the earliest stages are less likely to have high SDS scores and experience depression again. In contrast, people with stubbornly pessimistic mindsets about the prevention and treatment of depression are at high risk of relapse in depression (47–49). In that case, it is difficult for them to improve D-Lit. Our findings may encourage further research to explore the relationship between the changes in D-Lit before and after patients experience depression and the recurrence rate of depression.

Additionally, our study showed a high prevalence of depression (including mild, moderate, and severe types) among Chinese adults during COVID-19, suggesting a need for public attention to depression. When the present study was conducted, China still adhered to the strict zero-case policy toward COVID-19. The COVID-19 infodemic, social isolation, and social and academic disruption have been reported to induce chronic anxiety and depressive symptoms, thereby devastating public mental health (42, 50, 51). For those with these symptoms, we recommended acquiring knowledge concerning the recognition and management of depression, which has been shown to be effective in enhancing resilience to depressive symptoms (36). Furthermore, proactively seeking social support from appropriate sources, such as family and friends, has been found to play a critical role in alleviating depressive symptoms (52).

To date, a few cross-sectional studies have directly or indirectly examined the relationship between depression literacy and depressive status and have shown that low depression literacy was linked with the increased risks of depression. However, very few studies have explored the predictive role of participants' initial depression literacy on the later development and progression of depressive mood. We believed that the current study is among the first to shed light on this relationship in a longitudinal manner with multiple cross-sectional analyses. Furthermore, we explored the association between depression literacy and participants' proactivity regarding protective behaviors. Overall, depression literacy, depression, and protective behaviors appeared to be highly interconnected.

Given that, as was shown, depression literacy can play a crucial role in preventing depressive symptoms. Some recommendations for enhancing depression literacy and awareness of depressive symptoms in the general population are structured in Table 4.

**Table 4:** Suggestions for strengthening public depression literacy and awareness of depressive symptoms.

1. School-based curriculum can be a great source of depression literacy for college students (18).
2. Standard literacy education on depression stigma and mental help-seeking was shown its effectiveness in improving D-Lit in the general population, especially for patients with limited D-Lit to combat depression (45, 53).
3. Dissemination of evidence-based information *via* depression apps helps raise awareness of depression among the general population (54).
4. Multi-component community campaigns through official websites, media advertisements, and community forums can be beneficial interventions to promote depression literacy in adults (55).

### 4.1. Limitations

The current study has several limitations. First, the sample size needs to be larger; meanwhile, the targeted subjects were limited to Chinese adults. Furthermore, the present policy regarding COVID-19 in China differs from that in most other countries (especially Western countries), thereby limiting the generalizability of this study. Extension of the research to other countries and to adolescents should be considered. Second, although three cross-sectional analyses in a longitudinal manner were conducted in this study, causal inferences between depression literacy, protective behaviors, and depression are restricted. Third, the Zung SDS was utilized as a tool for appraising depressive status rather than clinical diagnoses by psychiatrists. This might lead to bias and therefore calls for more rigorous and robust approaches in future studies, where applicable.

## 5. Conclusion

The main purpose of this study was to explore the relationship between D-Lit and the later development and progression of depressive mood in Chinese adult social media users during COVID-19 and that between D-Lit and proactivity in protective behaviors against low mood. The results suggested that low depression literacy can be associated with exacerbated development and progression of depressive mood, which can eventually lead to depression if not appropriately and timely controlled. Furthermore, this study indirectly revealed the positive effect of protective behaviors on depressive mood. Increasing the level of D-Lit needs to be taken into consideration as an essential strategy for preventing and reversing depressive symptoms. Correct information about the recognition and management of depression needs to be disseminated. In the future, we encourage further research to explore practical and efficient ways to improve public depression literacy.

## Data availability statement

The raw data supporting the conclusions of this article will be made available by the authors, without undue reservation.

## Ethics statement

Ethical review and approval was not required for the study on human participants in accordance with the local legislation and institutional requirements. The patients/participants provided their written informed consent to participate in this study.

## Author contributions

DS, SL, and RX: conceptualization, methodology, software, validation, formal analysis, and writing the original draft. JHu and YW: investigation, resources, data curation, and writing the original draft. YZ, JHa, SS, and ZD: resources, writing, reviewing, editing, and visualization. DS: supervision and project administration. SH and YS: final revisions. All authors contributed to the article and approved the submitted version.
