# Correction: Low depression literacy exacerbates the development and progression of depressive mood in Chinese adult social media users during COVID-19: a 3-month observational online questionnaire-based study with multiple cross-sectional analyses

**DOI:** 10.3389/fpubh.2026.1926107

**Published:** 2026-08-17

**Authors:** Dan Shan, Shaoyang Li, Ruichen Xu, Jingtao Huang, Yi Wang, Yuandian Zheng, Shanshan Huang, Yuming Song, Junchu Han, Sayaka Suto, Zhihao Dai

**Affiliations:** 1 COVID-19 Response Social Organization Collaboration Network, Qujing, China; 2 Faculty of Science, The Hong Kong Polytechnic University, Kowloon, Hong Kong SAR, China; 3 Department of Integrative Biology, University of Wisconsin-Madison, Madison, WI, United States; 4 School of Basic Medical Science, Capital Medical University, Beijing, China; 5 China-Japan Friendship Clinical School, Beijing University of Chinese Medicine, Beijing, China; 6 Department of Medical Imaging, Qujing Second People's Hospital of Yunnan Province, Qujing, China; 7 School of Medical Imaging, Hebei Medical University, Shijiazhuang, China; 8 Department of Human Development, Columbia University, New York, NY, United States; 9 School of Medicine, Royal College of Surgeons, Ireland, University of Medicine and Health Sciences, Dublin, Ireland

**Keywords:** Chinese adults, COVID-19, depression, depression literacy, mental health

Affiliation “COVID-19 Response Social Organization Collaboration Network, Qujing, China” was erroneously given as “Department of Biobehavioral Sciences, Columbia University, New York, NY, United States” for authors Dan Shan and Yuandian Zheng.

In the published article, there was a wording error in the description of the event period in the questionnaire contents. The original wording referred to “September 2021 to September 2022,” which covered a period of strict COVID-19 control measures and recurrent outbreaks in China. However, this wording could be misunderstood as referring to the survey administration period. To improve clarity and align the questionnaire item with the study-period description, the wording has been corrected. This correction clarifies the questionnaire item and does not affect the final analytic sample size, statistical analyses, results, or conclusions of the article.

A correction has been made to the section **2. Materials and methods**, *2.2. Questionnaire contents*, paragraph 2, bulletpoint 7.

The previous version appeared as:

“Did you experience any personal events during the survey period of September 2021 to September 2022 that led to a significantly acute deterioration in your mood?”

The corrected version appears below:

“Did you experience any personal events during the survey period that led to a significantly acute deterioration in your mood?”

In the published article, there was a reporting error in the sex distribution of the final analytic sample in the Methods section (originally displayed as: 261 women and 192 men). The correct sex distribution was 251 women and 237 men, as reported in the Results section and Table 2. This correction does not affect the final sample size, statistical analyses, results, or conclusions of the article.

A correction has been made to the section **2. Materials and methods**, *2.2. Questionnaire contents*, paragraph 3.

The sentences previously appeared as:

“Eventually, a valid sample of 488 participants was analyzed collectively (261 women and 192 men; mean age = 32.62 years, SD = 6.71 years; age range: 18–49 years). The effective response rate was 81.3%.”

The corrected version appears below:

“Eventually, a valid sample of 488 participants was analyzed collectively (251 women and 237 men; mean age = 32.62 years, SD = 6.71 years; age range: 18–49 years). The effective response rate was 81.3%.”

The original version of this article has been updated.

